# Machine Learning to Identify Physician Actions Associated with Patient Experience of Compassion

**DOI:** 10.1007/s11606-025-09914-8

**Published:** 2025-10-21

**Authors:** Clifford M. Marks, Patrice Baptista, Cameron Gaines, Christopher W. Jones, Lauren Remboski, Andrew Nyce, Amanda M. Scudder, Adrian D. Haimovich, Nathan I. Shapiro, Stephen Trzeciak, Brian W. Roberts

**Affiliations:** 1 The Department of Emergency Medicine, Beth Israel Deaconess Medical Center, Boston, MA USA; 2 The Department of Emergency Medicine, Cooper University Health Care (CUHC), Cooper Medical School of Rowan University (CMSRU), NJ Camden, USA; 3 The Department of Medicine, CUHC/CMSRU, NJ Camden, USA; 4 Center for Humanism, Cooper Medical School of Rowan University, Camden, NJ USA

**Keywords:** compassion, empathy, physician–patient relationship

## Abstract

**Background:**

Physician compassion is associated with improvement in a variety of patient outcomes, but it remains unclear which individual physician behaviors affect patients’ experience of compassion.

**Objective:**

To determine which physician behaviors are most associated with patients’ experience of compassion.

**Design:**

We conducted a cross-sectional study at two urban academic emergency departments (ED) from September 2023 to May 2024. Participants completed questionnaires with a previously validated 5-item compassion measure and questions about whether the patient’s physician exhibited any of 27 behaviors previously proposed to be associated with compassion.

**Participants:**

We enrolled adult (age 18 years or older) patients presenting to each ED.

**Main Measures:**

The primary outcome was patient experience of physician compassion using the 5-item compassion measure. We used the machine learning algorithm LASSO to identify the group of actions that best predict the 5-item compassion measure. We performed exploratory analyses using linear regression with interaction terms to test for differences by patient race (White vs Black) and gender.

**Results:**

A total of 1025 patients completed the questionnaire (717 from site 1; 308 from site 2). The action with the strongest association with greater compassion was “Listen carefully to what you had to say” [β = 5.67 (95% CI 3.36 to 7.98)]. The LASSO algorithm identified nine actions as the best predictors, and this model explained roughly a third of variation in compassion scores: *r*^2^ = 0.34 in the derivation cohort and *r*^2^ = 0.32 in the validation cohort. Three behaviors had a stronger association with compassion scores among women (compared to men) and five behaviors among Black (compared to White) individuals.

**Conclusions and Relevance:**

These results show specific physician behaviors are associated with patients’ experience of compassion. Further research is needed to develop interventions to foster these behaviors and test prospectively whether they improve patients’ experience of compassion.

**Supplementary Information:**

The online version contains supplementary material available at 10.1007/s11606-025-09914-8.

## INTRODUCTION

Compassion is an integral part of health care but has only recently become the subject of rigorous quantitative research. Defined as an emotional response to another’s pain or suffering involving an authentic desire to help, compassion overlaps but differs from empathy, which is typically defined as understanding another’s emotions or the emotional experience of another’s feelings.^1^ Compassion involves taking action to alleviate another’s suffering and has been associated with improved symptoms of post-traumatic stress disorder (PTSD), reduced pain severity, greater patient adherence to treatment, and better glycemic control.^2–6^ It was been proposed that compassion can improve outcomes through gaining patients’ trust in their clinician.^7,8^

Researchers have tested a variety of interventions designed to inculcate compassion through videos, roleplay with feedback, and even changes to the physical environment.^9^ They have been aided by the development of multiple patient-reported outcome measures for compassion, a departure from previous scales in which providers rated themselves—a less useful indicator of how patients experience compassion than patients’ own perceptions.

Yet even with new tools devoted to compassion, efforts to teach this valuable facet of patient care have yielded mixed results. This partly reflects the fact that we still have little idea which physician behaviors affect a patient’s experience of compassion. Absent such knowledge, attempts to instill compassion will likely fall short of their potential. We aimed to fill this gap in the compassion literature. Our primary objectives were (1) to identify which physician behaviors are most associated with patient-reported compassion as measured by a previously validated compassion scale, and (2) to identify which physician behaviors are most associated with patient-reported compassion in patient subgroups divided by gender and race.

## METHODS

### Setting

We conducted this cross-sectional study in two urban academic emergency departments in the USA—Beth Israel Deaconess Medical Center (BIDMC), Boston, Massachusetts and Cooper University Hospital (CUH), Camden, New Jersey—from September 2023 to May 2024. This study is reported in accordance with the Strengthening the Reporting of Observational Studies in Epidemiology (STROBE) Statement (Supplemental Table 1)^10^ and the Checklist for Reporting Of Survey Studies (CROSS) (Supplemental Table 1).^11^

### Human Ethics and Consent to Participate

Each hospital’s Institutional Review Board approved the study, and all subjects provided written informed consent prior to participation. Subjects were not compensated for their time.

### Study Population

We enrolled a convenience sample of adult ED patients at each site. Inclusion criteria were as follows: (1) age 18 years or older; (2) presenting as a patient to the ED; and (3) English or Spanish speaking. Exclusion criteria included the following: (1) having an acute psychiatric emergency; (2) inability to participate (i.e., history of dementia, critically ill); (3) previously participated in the study; and (4) prisoner.

### Data Collection

Research assistants approached potential subjects in the ED for enrollment after completion of care by the ED clinician (i.e., either time of hospital admission or discharge from the ED). After obtaining written informed consent, the research assistants gave the subjects a computer tablet with the research questionnaire in electronic form. The Research Electronic Data Capture (REDCap) survey distribution tool was used to administer the research questionnaire and capture responses directly into the research database.^12,13^ Research assistants were then instructed to leave the patients’ bedside to allow for privacy while filling out the questionnaire, unless the patient requested they stay to help, and to return in 15 min to collect the tablet.

Our group previously performed a systematic review of compassion training for physicians.^9^ Based on this review, we derived a list of 27 physician actions previously suggested to increase patient experience of compassion (SupplementalTable 3). The research questionnaire queried patients if the ED physician performed each action during the ED encounter. Subjects answered, “yes,” “no,” or “unsure” for each action. The questionnaire collected patient demographic information. Using a standardized data collection form, we collected ED length of stay, and disposition from the ED (discharge vs. observation/hospital admission) from the medical record.

### Primary Outcome Measure

**Figure 1:**
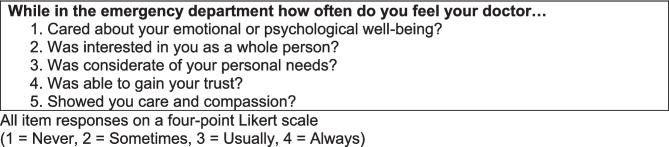
The 5-item compassion measure. All item responses on a four-point Likert scale (1 = Never, 2 = Sometimes, 3 = Usually, 4 = Always).

The primary outcome measure was patient experience of ED physician compassion using the 5-item compassion measure. This measure was previously psychometrically validated for use in the ED, outpatient, and inpatient settings in over 12,000 patients and consists of five items measured on a 4-point Likert scale (Fig. 1).^14–16^ The 5-item compassion measure was administered prior to questions pertaining to the actions and the items were administered in the order shown in Fig. 1 (there was no randomization). Scores for the five items were summed to obtain a composite compassion score. Potential scores range from 5 to 20, with higher scores indicating greater compassion. We entered all data into REDCap, and exported into Stata/SE 18.0 for Mac, StataCorp LP (College Station, TX, USA) for analysis.

### Data Analysis

We report continuous variables as median and interquartile range (IQR), and categorical variables as frequencies and percentages. We tested the internal reliability of the 5-item compassion measure using Cronbach’s alpha and display the full distribution of the composite compassion score in histogram form.

### Model Training

Data from BIDMC (site one) was used as the “training set” to identify the combination of actions that best predicted a higher 5-item compassion score. We performed separate pairwise univariable linear regression analyses to identify which actions were associated with the 5-item compassion score, with each action as a binary independent variable (yes vs. no/unsure) and the 5-item compassion measure as a continuous dependent variable. We used conservative robust standard errors to estimate the 95% CI to reduce the risk of type I error. We considered an action to be associated with the 5-item compassion score if the 95% CI for the beta coefficient did not cross zero. Given the potential for multicollinearity between measured actions, we used variance inflation factor (VIF) to test for high correlation between actions, which can produce unstable regression models. VIF < 5 indicates multicollinearity is not a problem. We used the machine learning algorithm LASSO to identify the group of actions that best predict the 5-item compassion measure. We entered the 5-item compassion measure as a continuous (ordinal) variable and performed a post hoc sensitivity analysis entering the 5-item compassion measure as a categorical variable: low (score < 15), moderate (score 15–19), and perfect (score 20) (See Supplemental Fig. 2 for details of LASSO models).

### Model Testing

Data from CUH (site two) was used as the “test set” to validate the final model in a separate cohort. We calculated the post-selection model fit for both sites separately and report them as *r*^2^. We then performed a linear regression model with the final actions as independent variables and the 5-item compassion measure as the dependent variable to determine the overall *r*^2^ across both cohorts and used Shorrocks-Shapley decomposition to determine each action’s relative contribution to the overall *r*^2^.

We tested if, among the validation cohort, a greater number of model actions reported being performed was associated with a greater 5-item compassion score using linear regression analysis and display the median compassion score by the number of actions performed using box plots. We also report the proportion of subjects with a perfect 5-item compassion score of 20 by the number of model actions reported being performed.

### Exploratory Analyses

We combined both data sets to test if the strength of the associations between individual actions and the 5-item compassion measure score differ by patient race (White vs. Black) and gender (male vs. female). We used separate pairwise multivariable linear regression models to test for an interaction between each action and race (reference: White) and gender (reference: male). We considered an interaction significant if the 95% CI (using robust standard errors) for the interaction term did not cross zero.

### Sample Size Calculation

There is no current method for sample size calculation for LASSO. LASSO can assess 1000 s of possible predictors at once, but the number of final selected predictors must be equal to or smaller than the sample size.^17^

## RESULTS

We administered the research questionnaire to 1075 patients (726 from site one and 349 from site two) and 1025 research questionnaires were completed (717 from site one and 308 from site two). The derivation cohort had a greater proportion of subjects with graduate-level education, a higher median annual income, and a greater proportion of subjects were discharged from the ED. The validation cohort was older, had a greater proportion of Black and Hispanic patients, and a greater proportion of subjects who did not attend college (Table 1).

**Table 1:** Patient Characteristics

**Variable**	**Derivation cohort** ***n* = 717**	**Validation cohort** ***n* = 308**
Age [years (IQR)]	49 (32 to 65)	57 (42 to 66)
Gender [*n* (%)]		
Male	269 (37.5)	126 (40.9)
Female	401 (55.9)	177 (57.5)
Transgender female	1 (0.1)	0
Transgender male	0	0
Non-binary	6 (0.8)	1 (0.3)
Not listed	2 (0.3)	0
Did not answer	38 (5.3)	4 (1.3)
Race [*n* (%)]		
White/Caucasian	444 (61.9)	180 (58.4)
Black/African American	149 (20.8)	82 (26.6)
Asian	43 (6.0)	4 (1.3)
Other	56 (7.8)	45 (14.6)
Unknown	43 (6.0)	0
Hispanic Ethnicity [*n* (%)]	62 (8.7)	65 (21.1)
Sexual orientation [*n* (%)]		
Heterosexual	523 (72.9)	235 (76.3)
Homosexual	35 (4.9)	15 (4.9)
Bisexual	37 (5.2)	11 (3.6)
Asexual	8 (1.1)	1 (0.3)
Not listed	16 (2.2)	22 (7.1)
Did not answer	98 (13.7)	24 (7.8)
Education [*n* (%)]		
Did not graduate high school	24 (3.4)	42 (13.6)
High school graduate or GED	143 (19.9)	106 (34.4)
Some college or 2-year degree	184 (25.7)	84 (27.3)
4-year college graduate	165 (23.0)	45 (14.6)
More than 4-year college degree	147 (20.5)	26 (8.4)
Did not answer	54 (7.5)	5 (1.6)
Reported income ($1000) [median (IQR)]	90 (45 to 160) *n* = 340	40 (20 to 78) *n* = 200
ED length of stay (hours) [median (IQR)]	9.1 (6.0–15.5)	10.1 (6.3–19.2)
Discharged from the ED	378 (52.8%)	111 (36.5%)

*ED* emergency department

The median (IQR) 5-item compassion measure score was 20 (18–20) and 20 (16–20) among the derivation and validation cohorts respectively. Sixty-six (9.2%) patients in the derivation cohort and 13 (4.2%) patients in the validation cohort were missing a response to at least one 5-item compassion measure item. The distributions of the 5-item compassion measure scores for each site are displayed in Supplemental Figs. 3 and 4. The 5-item compassion measure scores for each site ranged from 5 (lowest perceived compassion) to 20 (highest perceived compassion). The 5-item compassion measure had excellent internal reliability at both sites (derivation cohort Cronbach’s alpha = 0.92; validation cohort Cronbach’s alpha = 0.89).

Patient responses to whether the physician performed the 27 actions are displayed in Supplemental Table 3. The proportion of subjects reporting “yes” for each action performed was similar between sites. The most common action performed at both was “Introduce themselves” (95.4% and 96.4% in the derivation and validation cohorts respectively), and the least common action performed was “Sit down (versus stood up) while speaking with you” (27.9% and 39.6% in the derivation and validation cohorts respectively). Except for “Introduce themselves” [β = 0.86 (95% CI − 1.30 to 3.02)], all actions had a statistically significant positive association with the compassion score, meaning if patients reported the action was performed, they experienced greater compassion compared to when the action was not performed (Table 2). The action most strongly associated with higher levels of compassion was “Listen carefully to what you had to say” [β = 5.67 (95% CI 3.36 to 7.98)] followed by “Take your concerns seriously, instead of shrugging them off” [β = 4.72 (95% CI 3.36 to 6.07)]. We did not find evidence of multicollinearity between physician actions (all VIF < 2) (Supplemental Table 4).

**Table 2:** Pairwise Univariable Linear Regression Models with Physician Actions (Patient Reported Physician Performed the Action, “yes” versus “no/unsure”) as the Independent Variable and the 5-item Compassion Measure as the Dependent Variable among the Derivation Cohort

Actions	β coefficients	95% CI	*p*-value
Listen carefully to what you had to say	5.7	3.4 to 8.0	< 0.001
Take your concerns seriously, instead of shrugging them off	4.7	3.4 to 6.1	< 0.001
Keep focus on you and not get distracted	4.5	2.6 to 6.3	< 0.001
Make and keep eye contact with you	4.0	2.1 to 5.8	< 0.001
Face you when you were talking, instead of facing the other way	3.8	1.2 to 6.3	0.004
Speak in a calm or soothing tone	3.4	1.2 to 5.6	0.003
Act non-judgmental toward you	2.8	1.6 to 4.0	< 0.001
Come close (within 3 feet) when talking to you, instead of across the room	2.7	0.9 to 4.5	0.003
Respond to (versus ignoring) opportunities to show compassion	2.4	1.7 to 3.0	< 0.001
Communicate hope	2.3	1.5 to 3.0	< 0.001
Get to know you as a person	2.2	1.7 to 2.6	< 0.001
Listen more than they talked	2.2	1.4 to 2.9	< 0.001
Give you emotional support (for example, “I am here with you”)	2.0	1.5 to 2.6	< 0.001
Encourage you to express emotions	1.9	1.5 to 2.4	< 0.001
Understand how their words might affect you	1.8	1.2 to 2.4	< 0.001
Validate your emotions (for example, “This is hard for you- anyone would feel upset”)	1.7	1.2 to 2.2	< 0.001
Ask your treatment preferences, and make decisions with you, together	1.7	1.1 to 2.2	< 0.001
Ask how your daily functioning has been affected	1.6	1.1 to 2.1	< 0.001
Acknowledge that your personal experiences influence your needs	1.6	1.1 to 2.1	< 0.001
Grasp and explore clues that you need compassion	1.5	1.0 to 1.9	< 0.001
Express humor	1.4	0.9 to 1.8	< 0.001
Ask about your worries (for example, “What worries you the most?”)	1.3	0.9 to 1.8	< 0.001
Make “small talk”	1.2	0.8 to 1.7	< 0.001
Acknowledge and name your emotions (for example, “You seem sad”)	1.2	0.8 to 1.6	< 0.001
Give you supportive touch (for example, touch your shoulder)	1.0	0.6 to 1.5	< 0.001
Introduce themselves	0.9	-1.3 to 3.0	0.435
Sit down (versus stood up) while speaking with you	0.5	0.03 to 1.0	0.037

*CI* confidence interval

The primary LASSO algorithm identified nine actions as the best predictors and the sensitivity analysis identified five actions (all also identified by the primary model) (Table 3). The primary model was a good predictor of patient experience of compassion in the derivation cohort (*r*^2^ = 0.34) and in the validation cohort (*r*^2^ = 0.32). Using linear regression analysis, we found the overall *r*^2^ = 0.35 across both cohorts. “Take your concerns seriously, instead of shrugging them off,” provided the greatest contribution to the overall *r*^2^ (27.6%) (Table 3). These results suggest the nine actions identified predict about a third of the variation in patient experience of compassion. The sensitivity model with the compassion score as a categorical variable did not perform as well (derivation cohort *r*^2^ = 0.27; validation cohort *r*^2^ = 0.20).

**Table 3:** Physician Actions Identified by the LASSO Algorithm as the Best Predictors of Patient Experience of Compassion. Shorrocks-Shapley Decomposition of the Multivariable Linear Regression Model (with the 5-item Compassion Measure as the Dependent Variable) was used to Determine the Relative Contribution of Each Action to the *r*^2^ Across Both Cohorts (*r*^2^ = 0.35)

Action Did your doctor…	Shapley value (percent of *r*^2^)
Take your concerns seriously, instead of shrugging them off*	0.10 (27.6)
Get to know you as a person*	0.04 (12.0)
Listen carefully to what you had to say	0.03 (10.5)
Act non-judgmental toward you	0.04 (10.3)
Communicate hope	0.03 (9.1)
Keep focus on you and not get distracted	0.03 (8.7)
Encourage you to express emotions*	0.03 (8.4)
Respond to (versus ignoring) opportunities to show compassion*	0.03 (7.9)
Listen more than they talked*	0.02 (5.5)

^*^Identified by the sensitivity analysis

Supplemental Fig. 5 displays the distribution of the 5-item compassion score by the number of model actions performed among the validation cohort. Using linear regression, we found for every additional action performed the compassion score increased by one point [β = 1.04 (95% CI 0.83 to 1.25)]. No patients who reported 0–4 actions were performed had a perfect compassion score (*n* = 21). Among subjects who reported 5–8 actions were performed, 41.9% (62/148) reported a perfect compassion score, and among subjects who reported all nine actions were performed, 76.8% (76/99) reported a perfect compassion score (Supplemental Fig. 4).

**Figure 2:**
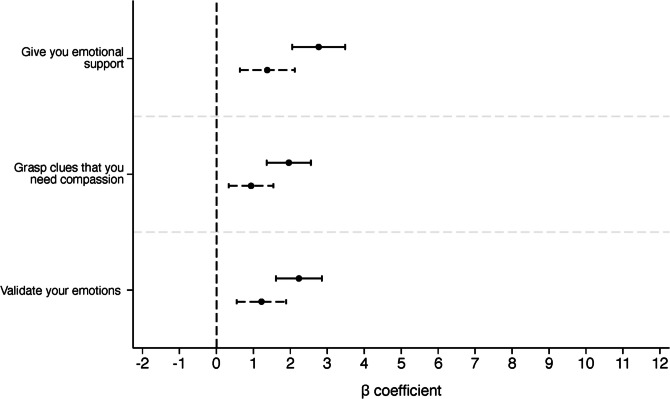
Results of the pairwise univariable linear regression models testing for interaction between physician actions (patient-reported physician performed the action, “yes” versus “no/unsure”) and patient sex (female versus male) for all items for which there was a statistically significant difference based on patient’s sex. β coefficients denote the difference in 5-item compassion measure score when the action is performed compared to when not performed. Horizontal lines are 95% confidence interval (solid = female, dashed = male).

**Figure 3:**
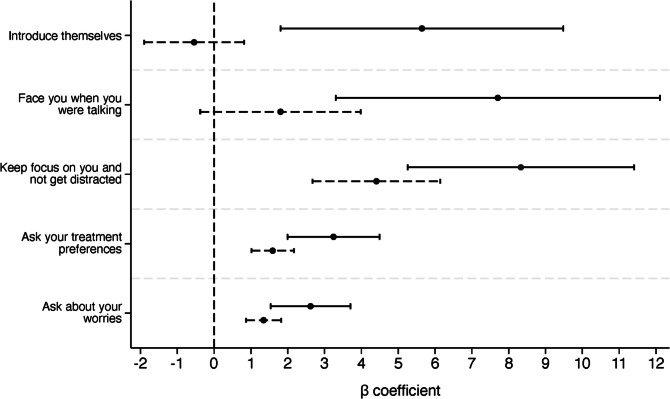
Results of the pairwise univariable linear regression models testing for interaction between physician actions (patient-reported physician performed the action, “yes” versus “no/unsure”) and patient race (Black versus non-Hispanic White) for all items for which there was a statistically significant difference based on patient’s race. β coefficients denote the difference in 5-item compassion measure score when the action is performed compared to when not performed. Horizontal lines are 95% confidence interval (solid = Black, dashed = non-Hispanic White).

In our exploratory analyses, we found three actions had a stronger association with patient experience of compassion among females compared to males: “Validate your emotions (for example, ‘This is hard for you—anyone would feel upset’),” “Grasp and explore clues that you need compassion,” and “Give you emotional support (for example, ‘I am here with you’) (Fig. 2). No actions had a stronger association with patient experience of compassion among males compared to females (Supplemental Figs. 6–7). We found five actions had a stronger association with patient experience of compassion among Black individuals compared to non-Hispanic White individuals: “Introduce themselves,” “Keep focus on you and not get distracted,” “Face you when you were talking, instead of facing the other way,” “Ask about your worries (for example, ‘What worries you the most?’)”, and “Ask your treatment preferences, and make decisions with you, together” (Fig. 3). No actions had a stronger association with patient experience of compassion among non-Hispanic White individuals compared to Black individuals (Supplemental Figs. 8–9).

## DISCUSSION

In this cross-sectional study, we sought to identify which physician actions were most associated with a higher level in patients’ experience of compassion. We found that nine actions predicted approximately one third of the variation in compassion experience. Our sensitivity analysis identified similar actions; however, the lower *r*^2^ may be due to loss of information and granularity when the 5-item compassion measure was treated as a categorical variable.^18^ These results remained consistent in a validation cohort that differed significantly from the derivation cohort, suggesting these results are generalizable to multiple patient populations. In the validation cohort, we also found an association between the number of actions performed and a greater experience of compassion. The actions identified center around staying focused on the patient and truly listening, getting to know the patient and understanding their concerns, communicating hope, and staying non-judgmental.

Both patients and clinicians consider compassion a vital aspect of high quality healthcare.^19^ Not only is compassionate care desired by patients, but greater compassion improves clinical outcomes.^1^ Specifically, in the emergency department greater patient experience of compassion is associated with decreased PTSD symptoms among patients experiencing a medical emergency,^6^ and decreased fear of stigma among patients with opioid use disorder.^20^ Thus, determining how to increase patient experience of compassion is of the utmost importance.

Our study identifies potential actions that if performed may increase patient experience of compassion. Prior research has focused on physician and nurse communication. For example, AIDET (Acknowledge, Introduce, Duration, Explanation, and Thank you) improved patient experience scores when implemented with other hospital-wide interventions.^21^ However, the specific effects of AIDET remain unknown. In addition, multiple studies show that the compassion scale used here measures aspects of care distinct from those captured by patient experience surveys.^14–16^ Another proposed framework is trauma-informed care, which proposes clinicians consider how patients’ prior experiences influence how they may perceive and react to medical care.^22^ However, a recent study of trauma-informed care did not find improved patient-reported outcomes, suggesting it may not impact patients’ experience of compassion.^23^ Further, neither framework was developed specifically to improve patient experience of compassion.

The results of this study provide an initial framework for interventions to increase patient experience of compassion by, for example, testing whether training physicians to perform the nine identified actions improves patient experience of compassion and other clinical outcomes. While this study identified nine actions that best predict patient experience of compassion, all the proposed actions aside from “Introduce themselves” were associated with increased compassion. The latter result likely reflects the fact that nearly all physicians reportedly introduced themselves, decreasing the ability to compare this behavior to its absence. Further, we found “Introduce themselves” had a statistically significant association with patient experience of compassion among female patients and among Black patients (Fig. 2). Therefore, it is possible that all the actions could increase compassion. However, given time restraints in the ED, we aimed to identify a parsimonious and pragmatic set of behaviors that could be easily performed at the bedside. Our exploratory analyses found evidence that different demographic groups may prioritize different actions in their assessments of physician compassion. This suggests that demonstrating compassion may not be “one size fits all,” but might benefit from actions tailored to an individual. Future research is needed to identify if specific actions better increase patient experience of compassion among different cohorts, particularly marginalized or stigmatized groups who may be subject to inequalities in healthcare. Future qualitative research is also warranted to identify if actions not included in our study might augment patient experience of compassion and to identify actions that are associated with decreased compassion, which clinicians should avoid. Additional qualitative research could help identify how best to encourage providers to perform the identified beneficial actions.

This study has limitations to consider. First, given its observational design, we can only state association between physician actions and patient experience of compassion. Second, this study was only conducted in EDs, where there are typically no established patient-clinician relationships. Actions may differ in other settings where there are established relationships. Third, we had patients report if they remembered the physician performing the action, raising the risk of recall bias. Future studies could use direct observation or video ethnography to identify which actions are performed and correlate these with patients’ recall of the actions and experience of compassion. To reduce the risk of recall bias, we administered the research questionnaire immediately after completion of the patient-physician interaction while the patients were still in the ED. We also enrolled a convenience sample—patients who presented to the ED when research staff were not available may have a different experience of physician actions and compassion. However, the predictive ability of the actions remained consistent in a demographically dissimilar validation cohort, suggesting robustness and generalizability of our results. Finally, consistent with prior studies of patient experience,^14–16,20^ most compassion scores were clustered at or near the maximum possible score. However, LASSO analysis is robust to non-normally distributed data and our sensitivity analysis categorizing the 5-item measure gave consistent results.

## CONCLUSION

This study found that patients reported a higher experience of compassion when they reported their physician demonstrated traits such as staying focused and truly listening, getting to know them and understanding their concerns, communicating hope, and staying non-judgmental. The findings also suggest that different demographic groups may value different behaviors when assessing physician compassion. Further research is warranted to develop interventions aimed at increasing physician performance of these actions to improve patients’ experience of compassion and related clinical outcomes.

## Supplementary Information

Below is the link to the electronic supplementary material.

ESM 1 (DOCX 804 KB)

## Data Availability

Data will be made publicly available at Roberts, Brian (2025), “DBAD”, Mendeley Data, V1, 10.17632/tp5vd9k53s.1.
