# Supplementary material for: Machine Learning to Identify Physician Actions Associated with Patient Experience of Compassion
Source: J Gen Intern Med. 2025 Oct 21;41(10):2694–701. doi: 10.1007/s11606-025-09914-8 (PMC13421575; doi:10.1007/s11606-025-09914-8)
Supplement: Supplementary file 1 — (DOCX 804 KB) [file 11606_2025_9914_MOESM1_ESM.docx]

**Machine learning to identify physician actions associated with patient experience of compassion.**

**Supplemental Material**

Clifford M. Marks, MD, MBA^1^; Patrice Baptista, MD^2^; Cameron Gaines, BA^2^;

Christopher W. Jones, MD^2^; Lauren Remboski, MS^2^; Andrew Nyce, MD^2^; Amanda M. Scudder, MD^1^; Adrian D. Haimovich, MD, PhD^1^; Nathan I. Shapiro, MD, MPH^1^;

Stephen Trzeciak, MD, MPH^3,4^; Brian W. Roberts, MD, MSc^2,4^

1: The Department of Emergency Medicine, Beth Israel Deaconess Medical Center, Boston, Massachusetts, USA

2: The Department of Emergency Medicine, Cooper University Health Care (CUHC), Cooper Medical School of Rowan University (CMSRU), Camden, New Jersey, USA

3: The Department of Medicine, CUHC/CMSRU, Camden, New Jersey, USA

4: Center for Humanism, Cooper Medical School of Rowan University, Camden, NJ, USA

For Submission to *Journal of General Internal Medicine*

Address for correspondence:

Brian W. Roberts, MD, MSc

Department of Emergency Medicine

Cooper University Health Care

Education & Research Building, Second Floor

401 Haddon Avenue

Camden, NJ 08103

roberts-brian-w@cooperhealth.edu

**Supplemental Table 1**: Strengthening the Reporting of Observational Studies in Epidemiology (STROBE) Statement checklist.

|  | Item No | Recommendation |
| --- | --- | --- |
| **Title and abstract** | 1 | (*a*) Indicate the study’s design with a commonly used term in the title or the abstract. Page 3 |
|  |  | (*b*) Provide in the abstract an informative and balanced summary of what was done and what was found. Page 3 |
| Introduction | | |
| Background/rationale | 2 | Explain the scientific background and rationale for the investigation being reported. Page 4 |
| Objectives | 3 | State specific objectives, including any prespecified hypotheses. Page 4 |
| Methods | | |
| Study design | 4 | Present key elements of study design early in the paper. Pages 5-9 |
| Setting | 5 | Describe the setting, locations, and relevant dates, including periods of recruitment, exposure, follow-up, and data collection. Page 5 |
| Participants | 6 | (*a*) Give the eligibility criteria, and the sources and methods of selection of participants. Describe methods of follow-up. Page 5 |
|  |  | (*b*) For matched studies, give matching criteria and number of exposed and unexposed. N/A |
| Variables | 7 | Clearly define all outcomes, exposures, predictors, potential confounders, and effect modifiers. Give diagnostic criteria, if applicable. Pages 5-9 |
| Data sources/ measurement | 8* | For each variable of interest, give sources of data and details of methods of assessment (measurement). Describe comparability of assessment methods if there is more than one group. Pages 5-9 |
| Bias | 9 | Describe any efforts to address potential sources of bias. Pages 5-9, 15 |
| Study size | 10 | Explain how the study size was arrived at. Page 10 |
| Quantitative variables | 11 | Explain how quantitative variables were handled in the analyses. If applicable, describe which groupings were chosen and why. Pages 7-9 |
| Statistical methods | 12 | (*a*) Describe all statistical methods, including those used to control for confounding. Pages 7-9 |
|  |  | (*b*) Describe any methods used to examine subgroups and interactions. Page 9 |
|  |  | (*c*) Explain how missing data were addressed. Pages 10-11 |
|  |  | (*d*) If applicable, explain how loss to follow-up was addressed. N/A |
|  |  | (*e*) Describe any sensitivity analyses. N/A |
| Results | | |
| Participants | 13* | (a) Report numbers of individuals at each stage of study—eg numbers potentially eligible, examined for eligibility, confirmed eligible, included in the study, completing follow-up, and analysed. Figure 1 |
|  |  | (b) Give reasons for non-participation at each stage. Figure 1 |
|  |  | (c) Consider use of a flow diagram. Figure 1 |
| Descriptive data | 14* | (a) Give characteristics of study participants (eg demographic, clinical, social) and information on exposures and potential confounders. Table 1 |
|  |  | (b) Indicate number of participants with missing data for each variable of interest. Table 1 |
|  |  | (c) Summarise follow-up time (eg, average and total amount). N/A |
| Outcome data | 15* | Report numbers of outcome events or summary measures over time. N/A |
| Main results | 16 | (*a*) Give unadjusted estimates and, if applicable, confounder-adjusted estimates and their precision (eg, 95% confidence interval). Make clear which confounders were adjusted for and why they were included. Tables 2 and 3 |
|  |  | (*b*) Report category boundaries when continuous variables were categorized. N/A |
|  |  | (*c*) If relevant, consider translating estimates of relative risk into absolute risk for a meaningful time period. N/A |
| Other analyses | 17 | Report other analyses done—eg analyses of subgroups and interactions, and sensitivity analyses. Figures 1 and 2 |
| Discussion | | |
| Key results | 18 | Summarise key results with reference to study objectives. Pages 10-12 |
| Limitations | 19 | Discuss limitations of the study, taking into account sources of potential bias or imprecision. Discuss both direction and magnitude of any potential bias. Page 15 |
| Interpretation | 20 | Give a cautious overall interpretation of results considering objectives, limitations, multiplicity of analyses, results from similar studies, and other relevant evidence. Pages 13-15 |
| Generalisability | 21 | Discuss the generalisability (external validity) of the study results. Page 15 |
| Other information | | |
| Funding | 22 | Give the source of funding and the role of the funders for the present study and, if applicable, for the original study on which the present article is based. Title Page |

**Supplemental Table 2:** Checklist for Reporting Of Survey Studies (CROSS)

| **Section/topic** | **Item** | **Item description** | **Reported on page #** |
| --- | --- | --- | --- |
| **Title and abstract** | | |  |
| Title and abstract | 1a | State the word “survey” along with a commonly used term in title or abstract to introduce the study’s design. | 1 |
|  | 1b | Provide an informative summary in the abstract, covering background, objectives, methods, findings/results, interpretation/discussion, and conclusions. | 1 |
| **Introduction** | | |  |
| Background | 2 | Provide a background about the rationale of study, what has been previously done, and why this survey is needed. | 5 |
| Purpose/aim | 3 | Identify specific purposes, aims, goals, or objectives of the study. | 5 |
| **Methods** | | |  |
| Study design | 4 | Specify the study design in the methods section with a commonly used term (e.g., cross-sectional or longitudinal). | 6 |
|  | 5a | Describe the questionnaire (e.g., number of sections, number of questions, number and names of instruments used). | 5-6, Supp. Table 3, Supp. Figure 1 |
| Data collection methods | 5b | Describe all questionnaire instruments that were used in the survey to measure particular concepts. Report target population, reported validity and reliability information, scoring/classification procedure, and reference links (if any). | 7 |
|  | 5c | Provide information on pretesting of the questionnaire, if performed (in the article or in an online supplement). Report the method of pretesting, number of times questionnaire was pre-tested, number and demographics of participants used for pretesting, and the level of similarity of demographics between pre-testing participants and sample population. | 7 |
|  | 5d | Questionnaire if possible, should be fully provided (in the article, or as appendices or as an online supplement). | Supp. Table 3, Supp. Figure 1 |
| Sample characteristics | 6a | Describe the study population (i.e., background, locations, eligibility criteria for participant inclusion in survey, exclusion criteria). | 6 |
|  | 6b | Describe the sampling techniques used (e.g., single stage or multistage sampling, simple random sampling, stratified sampling, cluster sampling, convenience sampling). Specify the locations of sample participants whenever clustered sampling was applied. | 6 |
|  | 6c | Provide information on sample size, along with details of sample size calculation. | 10 |
|  | 6d | Describe how representative the sample is of the study population (or target population if possible), particularly for population-based surveys. |  |
| Survey  administration | 7a | Provide information on modes of questionnaire administration, including the type and number of contacts, the location where the survey was conducted (e.g., outpatient room or by use of online tools, such as SurveyMonkey). | 6-7 |
|  | 7b | Provide information of survey’s time frame, such as periods of recruitment, exposure, and follow-up days. | 6-7 |
|  | 7c | Provide information on the entry process:  –>For non-web-based surveys, provide approaches to minimize human error in data entry.  –>For web-based surveys, provide approaches to prevent “multiple participation” of participants. | 6-7 |
| Study preparation | 8 | Describe any preparation process before conducting the survey (e.g., interviewers’ training process, advertising the survey). | 6-7 |
| Ethical considerations | 9a | Provide information on ethical approval for the survey if obtained, including informed consent, institutional review board [IRB] approval, Helsinki declaration, and good clinical practice [GCP] declaration (as appropriate). | 6 |
|  | 9b | Provide information about survey anonymity and confidentiality and describe what mechanisms were used to protect unauthorized access. | 6-7 |
| Statistical  analysis | 10a | Describe statistical methods and analytical approach. Report the statistical software that was used for data analysis. | 7-9, Supp. Figure 2 |
|  | 10b | Report any modification of variables used in the analysis, along with reference (if available). | N/A |
|  | 10c | Report details about how missing data was handled. Include rate of missing items, missing data mechanism (i.e., missing completely at random [MCAR], missing at random [MAR] or missing not at random [MNAR]) and methods used to deal with missing data (e.g., multiple imputation). | 10-11 |
|  | 10d | State how non-response error was addressed. | 6-7 |
|  | 10e | For longitudinal surveys, state how loss to follow-up was addressed. | N/A |
|  | 10f | Indicate whether any methods such as weighting of items or propensity scores have been used to adjust for non-representativeness of the sample. | N/A |
|  | 10g | Describe any sensitivity analysis conducted. | N/A |
| **Results** | | |  |
| Respondent characteristics | 11a | Report numbers of individuals at each stage of the study. Consider using a flow diagram, if possible. | Page 10 |
|  | 11b | Provide reasons for non-participation at each stage, if possible. | Page 10 |
|  | 11c | Report response rate, present the definition of response rate or the formula used to calculate response rate. | Page 10 |
|  | 11d | Provide information to define how unique visitors are determined. Report number of unique visitors along with relevant proportions (e.g., view proportion, participation proportion, completion proportion). | Page 10 |
| Descriptive  results | 12 | Provide characteristics of study participants, as well as information on potential confounders and assessed outcomes. | Table 1 |
| Main findings | 13a | Give unadjusted estimates and, if applicable, confounder-adjusted estimates along with 95% confidence intervals and p-values. | Table 2 |
|  | 13b | For multivariable analysis, provide information on the model building process, model fit statistics, and model assumptions (as appropriate). | 11-12, Supp. Figure 2 |
|  | 13c | Provide details about any sensitivity analysis performed. If there are considerable amount of missing data, report sensitivity analyses comparing the results of complete cases with that of the imputed dataset (if possible). | N/A |
| **Discussion** | | |  |
| Limitations | 14 | Discuss the limitations of the study, considering sources of potential biases and imprecisions, such as non-representativeness of sample, study design, important uncontrolled confounders. | 15 |
| Interpretations | 15 | Give a cautious overall interpretation of results, based on potential biases and imprecisions and suggest areas for future research. | 13-15 |
| Generalizability | 16 | Discuss the external validity of the results. | 15 |
| **Other sections** | | |  |
| Role of funding source | 17 | State whether any funding organization has had any roles in the survey’s design, implementation, and analysis. | Title page |
| Conflict of interest | 18 | Declare any potential conflict of interest. | Acknowledgements |
| Acknowledgements | 19 | Provide names of organizations/persons that are acknowledged along with their contribution to the research. | Title page |

**Supplemental Table 3**: Subject responses to physician actions [n (%)]. Bold font denotes actions included in the final model.

| **Action*** | **Derivation (n = 717)** | | | | **Validation (n = 308)** | | | |
| --- | --- | --- | --- | --- | --- | --- | --- | --- |
|  | Yes | No | Unsure | Missing | Yes | No | Unsure | Missing |
| 1 | 684 (95.4) | 4 (0.6) | 17 (2.4) | 12 (1.7) | 297 (96.4) | 6 (2.0) | 2 (0.7) | 3 (1.0) |
| 2 | 198 (27.6) | 453 (63.2) | 51 (7.1) | 15 (2.1) | 122 (39.6) | 160 (52.0) | 16 (5.2) | 10 (3.3) |
| 3 | 676 (94.3) | 12 (1.7) | 17 (2.4) | 12 (1.7) | 286 (92.9) | 8 (2.6) | 8 (2.6) | 6 (2.0) |
| **4** | **667 (93.0)** | **20 (2.8)** | **18 (2.5)** | **12 (1.7)** | **288 (93.5)** | **10 (3.3)** | **4 (1.3)** | **6 (2.0)** |
| 5 | 680 (94.8) | 10 (1.4) | 13 (1.8) | 14 (2.0) | 290 (94.2) | 10 (3.3) | 1 (0.3) | 7 (2.3) |
| 6 | 670 (93.4) | 15 (2.1) | 18 (2.5) | 14 (2.0) | 282 (91.6) | 15 (4.9) | 4 (1.3) | 7 (2.3) |
| **7** | **673 (93.9)** | **16 (2.2)** | **13 (1.8)** | **15 (2.1)** | **292 (94.8)** | **8 (2.6)** | **4 (1.3)** | **4 (1.3)** |
| **8** | **565 (78.8)** | **42 (5.9)** | **91 (12.7)** | **19 (2.7)** | **226 (73.4)** | **44 (14.3)** | **27 (8.8)** | **11 (3.6)** |
| 9 | 675 (94.1) | 15 (2.1) | 11 (1.5) | 16 (2.2) | 285 (92.5) | 8 (2.6) | 4 (1.3) | 11 (3.6) |
| 10 | 302 (42.1) | 299 (41.7) | 95 (13.3) | 21 (2.9) | 156 (50.7) | 118 (38.3) | 19 (6.2) | 15 (4.9) |
| 11 | 350 (48.8) | 246 (34.3) | 99 (13.8) | 22 (3.1) | 186 (60.4) | 97 (31.5) | 15 (4.9) | 10 (3.3) |
| **12** | **402 (56.1)** | **187 (26.1)** | **103 (14.4)** | **25 (3.5)** | **199 (64.6)** | **76 (24.7)** | **21 (6.8)** | **12 (3.9)** |
| **13** | **635 (88.6)** | **25 (3.5)** | **34 (4.7)** | **23 (3.2)** | **274 (89.0)** | **14 (4.6)** | **9 (2.9)** | **11 (3.6)** |
| **14** | **405 (56.5)** | **135 (18.8)** | **144 (20.1)** | **33 (4.6)** | **182 (59.1)** | **74 (24.0)** | **36 (11.7)** | **16 (5.2)** |
| 15 | 304 (42.4) | 208 (29.0) | 172 (24.0) | 33 (4.6) | 162 (52.6) | 100 (32.5) | 27 (8.8) | 19 (6.2) |
| 16 | 419 (58.4) | 139 (19.4) | 121 (16.9) | 38 (5.3) | 195 (63.3) | 68 (22.1) | 25 (8.1) | 20 (6.4) |
| 17 | 376 (52.4) | 123 (17.2) | 172 (24.0) | 46 (6.2) | 168 (54.6) | 72 (23.4) | 47 (15.3) | 21 (6.8) |
| **18** | **482 (69.3)** | **81 (11.3)** | **105 (14.6)** | **49 (6.8)** | **228 (74.0)** | **40 (13.0)** | **22 (7.1)** | **18 (5.8)** |
| 19 | 450 (62.8) | 115 (16.0) | 106 (14.8) | 46 (6.4) | 228 (74.0) | 48 (15.6) | 15 (4.9) | 17 (5.5) |
| 20 | 438 (61.1) | 163 (22.7) | 83 (11.6) | 33 (4.6) | 207 (67.2) | 68 (22.1) | 13 (4.2) | 20 (6.5) |
| 21 | 359 (50.1) | 215 (30.0) | 97 (13.5) | 46 (6.4) | 176 (57.1) | 101 (32.8) | 11 (3.6) | 20 (6.5) |
| 22 | 428 (59.7) | 154 (21.5) | 88 (12.3) | 47 (6.6) | 218 (70.8) | 55 (17.9) | 14 (4.6) | 21 (6.9) |
| **23** | **526 (73.4)** | **77 (10.7)** | **68 (9.5)** | **46 (6.4)** | **247 (80.2)** | **27 (8.8)** | **15 (4.9)** | **19 (6.2)** |
| 24 | 467 (65.1) | 60 (8.4) | 137 (19.1) | 53 (7.4) | 214 (69.5) | 35 (11.4) | 33 (10.7) | 26 (8.4) |
| **25** | **600 (83.7)** | **37 (5.2)** | **39 (5.4)** | **41 (5.7)** | **246 (79.9)** | **31 (10.1)** | **11 (3.6)** | **20 (6.5)** |
| 26 | 430 (60.0) | 82 (11.4) | 141 (19.7) | 64 (8.9) | 216 (70.1) | 37 (12.0) | 33 (10.7) | 22 (7.1) |
| 27 | 253 (35.3) | 307 (42.8) | 95 (13.3) | 62 (8.7) | 169 (54.9) | 98 (31.8) | 20 (6.5) | 21 (6.8) |

*Did you doctor…

1. Introduce themselves

2. Sit down (versus stood up) while speaking with you

3. Make and keep eye contact with you

**4. Keep focus on you and not get distracted**

5. Face you when you were talking, instead of facing the other way

6. Come close (within 3 feet) when talking to you, instead of across the room

**7. Listen carefully to what you had to say**

**8. Listen more than they talked**

9. Speak in a calm or soothing tone

10. Make "small talk"

11. Express humor

**12. Get to know you as a person**

**13. Take your concerns seriously, instead of shrugging them off**

**14. Encourage you to express emotions**

15. Acknowledge and name your emotions (for example, "You seem sad")

16. Validate your emotions (for example, "This is hard for you- anyone would feel upset")

17. Grasp and explore clues that you need compassion

**18. Respond to (versus ignoring) opportunities to show compassion**

19. Give you emotional support (for example, "I am here with you")

20. Ask how your daily functioning has been affected

21. Ask about your worries (for example, "What worries you the most?")

22. Ask your treatment preferences, and make decisions with you, together

**23. Communicate hope**

24. Understand how their words might affect you

**25. Act non-judgmental toward you**

26. Acknowledge that your personal experiences influence your needs

27. Give you supportive touch (for example, touch your shoulder)

**Supplemental Table 4**: Test of multicollinearity using variance inflation factor (VIF). Bold font denotes actions included in the final model.

| **Actions** | **VIF** |
| --- | --- |
|  |  |
| Introduce themselves | 1.2 |
| Sit down (versus stood up) while speaking with you | 1.1 |
| Make and keep eye contact with you | 1.4 |
| **Keep focus on you and not get distracted** | **1.7** |
| Face you when you were talking, instead of facing the other way | 1.8 |
| Come close (within 3 feet) when talking to you, instead of across the room | 1.5 |
| **Listen carefully to what you had to say** | **1.4** |
| **Listen more than they talked** | **1.2** |
| Speak in a calm or soothing tone | 1.5 |
| Make "small talk" | 1.6 |
| Express humor | 1.6 |
| **Get to know you as a person** | **1.5** |
| **Take your concerns seriously, instead of shrugging them off** | **1.5** |
| **Encourage you to express emotions** | **1.6** |
| Acknowledge and name your emotions (for example, "You seem sad") | 1.7 |
| Validate your emotions (for example, "This is hard for you- anyone would feel upset") | 1.8 |
| Grasp and explore clues that you need compassion | 1.7 |
| **Respond to (versus ignoring) opportunities to show compassion** | **1.7** |
| Give you emotional support (for example, "I am here with you") | 1.8 |
| Ask how your daily functioning has been affected | 1.5 |
| Ask about your worries (for example, "What worries you the most?") | 1.6 |
| Ask your treatment preferences, and make decisions with you, together | 1.5 |
| **Communicate hope** | **1.5** |
| Understand how their words might affect you | 1.6 |
| **Act non-judgmental toward you** | **1.5** |
| Acknowledge that your personal experiences influence your needs | 1.7 |
| Give you supportive touch (for example, touch your shoulder) | 1.3 |

**Supplemental Figure 1**: 5-item compassion measure.


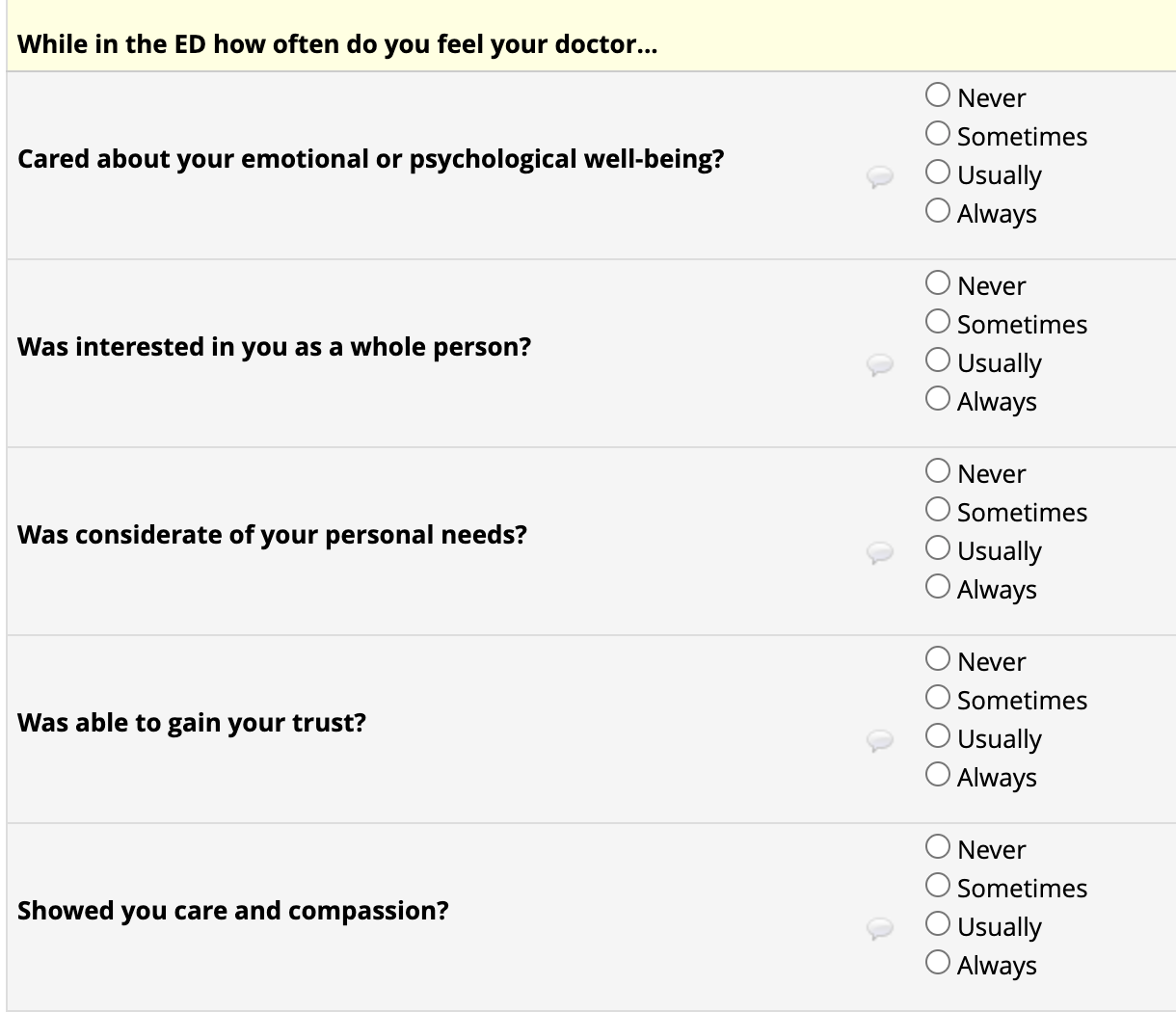


**Supplemental Figure 2**: Description of LASSO machine learning algorithm

LASSO is a method for selecting a set of variables that best predict an outcome. We entered all actions except for “Introduce themselves.” We did not include this action given less than 10 patients reported “no” for this action in the derivation cohort, resulting in a lack of variability. LASSO randomly divides the dataset into 10 folds. Each model is trained (i.e. estimated) on nine of the folds; then the derived model is applied to the hold-out fold and the out-of-sample predicted error is calculated. This process is repeated leaving out and using a different fold for validation each time until all 10 folds have been left out. The predicted error is averaged across the 10 hold-out folds. LASSO repeats this process using different models (i.e. different sets of variables) to identify the model with the minimal average out-of-sample prediction error (cross validation), while adding a model penalty that increases in value the more complex the model. This penalty causes LASSO to omit variables to prevent overfitting of the model. Thus, LASSO selects the model most suitable for making predictions in samples outside the dataset used for derivation. To identify the most parsimonious model we used the adaptive method for model selection. The adaptive method performs LASSO once, then calculates penalty weights from the coefficient estimates. These weights are used in a second LASSO to remove variables that may not be contributing substantially to the model. Of note, LASSO identifies a model that best predicts the outcome; it does not find a model that allows for interpreting estimated coefficients as effects. In other words, LASSO aims to identify the group of actions that when patients report they are performed by the physician best predict a greater experience of compassion; LASSO is not used to estimate the effects of individual actions on the experience of compassion.

We *a priori* planned to enter the 5-item compassion measure as a continuous variable into the LASSO model. Given the left skewness of the 5-item compassion measure we performed a *post hoc* sensitivity analysis entering the 5-item compassion measure as a categorical variable: low (score < 15), moderate (score 15-19), and perfect (score 20). We selected a cut point of 15 given this was most commonly a score of three for each item, suggesting moderate compassion across all items, while a score of 14 or less would require a score of two or less on the majority of items. Although a large proportion of subjects had a perfect score (score = 20), we did not perform an analysis entering the 5-item as a binary outcome (perfect vs. not perfect) as this would treat patients with a score of 5 (no compassion) and 19 (almost perfect compassion) the same, reducing granularity in prediction.

**Supplemental Figure 3**: Distribution of the physician 5-item compassion measure among the derivation cohort.

**Supplemental Figure 4**: Distribution of the physician 5-item compassion measure among the validation cohort.

**Supplemental Figure 5**: Distribution of 5-item compassion measure score by number of model actions performed among the validation cohort. Black horizontal line indicates median score. Gray box indicates interquartile range. Whiskers indicate upper and lower adjacent values*. Black circles indicate scores outside the upper and lower adjacent values.

*Lower adjacent value equals smallest value within 25^th^ percentile minus 1.5 times the interquartile range. Upper adjacent value equals the largest value within 75^th^ percentile plus 1.5 times the interquartile range.

**Supplemental Figure 6**: Results of the pairwise univariable linear regression models testing for an interaction between physician actions included in the final model (patient reported physician performed the action, “yes” versus “no/unsure”) and sex (female versus male). The 5-item compassion measure is the dependent variable. β coefficients denote the difference in 5-item compassion measure score when the action is performed compared to when not performed. Horizontal lines are 95% confidence interval (solid = female, dashed = male).

| Actions | Interaction term β Coefficients | 95% CI | p-value |
| --- | --- | --- | --- |
|  |  |  |  |
| Take your concerns seriously, instead of  shrugging them off | 1.9 | -0.5 to 4.2 | 0.116 |
| Act non-judgmental toward you | 1.5 | -0.4 to 3.4 | 0.123 |
| Respond to (versus ignoring) opportunities to  show compassion | 0.9 | -0.2 to 2.0 | 0.116 |
| Communicate hope | 0.8 | -0.5 to 2.2 | 0.231 |
| Encourage you to express emotions | 0.8 | -0.1 to 1.6 | 0.080 |
| Listen carefully to what you had to say | 0.7 | -2.9 to 4.4 | 0.698 |
| Get to know you as a person | 0.4 | -0.4 to 1.3 | 0.330 |
| Listen more than they talked | 0.3 | -1.0 to 1.5 | 0.678 |
| Keep focus on you and not get distracted | 0.1 | -3.3 to 3.5 | 0.963 |

CI, confidence interval. Interaction term β Coefficients denote the difference in the β Coefficients between females and males. Interaction term β Coefficients > 0 indicate the association between the physician action and 5-item compassion measure score are stronger among females compared to males (reference). Interaction term β Coefficients < 0 indicate the association between the physician action and 5-item compassion measure score are stronger among males compared to females.

**Supplemental Figure 7**: Results of the pairwise univariable linear regression models testing for an interaction between physician actions not included in the final model (patient reported physician performed the action, “yes” versus “no/unsure”) and sex (female versus male). The 5-item compassion measure is the dependent variable. β coefficients denote the difference in 5-item compassion measure score when the action is performed compared to when not performed. Horizontal lines are 95% confidence interval (solid = female, dashed = male).

| Actions | Interaction term β Coefficients | 95% CI | p-value |
| --- | --- | --- | --- |
|  |  |  |  |
| Introduce themselves | 4.2 | -0.7 to 9.1 | 0.090 |
| Make and keep eye contact with you | 2.0 | -1.0 to 5.0 | 0.183 |
| Speak in a calm or soothing tone | 1.7 | -2.1 to 5.5 | 0.383 |
| Give you emotional support (for example, "I am  here with you") | 1.4 | 0.4 to 2.4 | 0.008 |
| Grasp and explore clues that you need  compassion | 1.0 | 0.2 to 1.9 | 0.018 |
| Validate your emotions (for example, "This is hard  for you- anyone would feel upset") | 1.0 | 0.1 to 1.9 | 0.030 |
| Ask your treatment preferences, and make  decisions with you, together | 0.9 | -0.1 to 1.9 | 0.090 |
| Face you when you were talking, instead of facing  the other way | 0.8 | -3.2 to 4.9 | 0.690 |
| Acknowledge and name your emotions (for  example, "You seem sad") | 0.8 | 0.0 to 1.6 | 0.051 |
| Give you supportive touch (for example, touch  your shoulder) | 0.8 | 0.0 to 1.5 | 0.062 |
| Ask about your worries (for example, "What  worries you the most?") | 0.6 | -0.2 to 1.4 | 0.140 |
| Sit down (versus stood up) while speaking with you | 0.6 | -0.2 to 1.4 | 0.166 |
| Come close (within 3 feet) when talking to you,  instead of across the room | 0.5 | -2.0 to 3.1 | 0.685 |
| Make "small talk" | 0.5 | -0.3 to 1.3 | 0.187 |
| Acknowledge that your personal experiences  influence your needs | 0.5 | -0.5 to 1.5 | 0.335 |
| Understand how their words might affect you | 0.4 | -0.7 to 1.5 | 0.439 |
| Ask how your daily functioning has been affected | 0.4 | -0.6 to 1.4 | 0.409 |
| Express humor | 0.2 | -0.6 to 1.0 | 0.573 |

CI, confidence interval. Interaction term β Coefficients denote the difference in the β Coefficients between females and males. Interaction term β Coefficients > 0 indicate the association between the physician action and 5-item compassion measure score are stronger among females compared to males (reference). Interaction term β Coefficients < 0 indicate the association between the physician action and 5-item compassion measure score are stronger among males compared to females.

**Supplemental Figure 8**: Results of the pairwise univariable linear regression models testing for an interaction between physician actions included in the final model (patient reported physician performed the action, “yes” versus “no/unsure”) and race (Black versus non-Hispanic White). The 5-item compassion measure is the dependent variable. β coefficients denote the difference in 5-item compassion measure score when the action is performed compared to when not performed. Horizontal lines are 95% confidence interval (solid = Black, dashed = non-Hispanic White).

| Actions | Interaction term β Coefficients | 95% CI | p-value |
| --- | --- | --- | --- |
|  |  |  |  |
| Keep focus on you and not get distracted | 3.9 | 0.4 to 7.4 | 0.029 |
| Listen carefully to what you had to say | 3.5 | -0.6 to 7.5 | 0.091 |
| Take your concerns seriously, instead of  shrugging them off | 2.2 | -0.5 to 4.9 | 0.108 |
| Listen more than they talked | 1.5 | -0.3 to 3.4 | 0.099 |
| Communicate hope | 1.4 | -0.4 to 3.2 | 0.119 |
| Encourage you to express emotions | 1.0 | -0.3 to 2.2 | 0.119 |
| Get to know you as a person | 0.9 | -0.3 to 2.1 | 0.138 |
| Act non-judgmental toward you | 0.7 | -1.6 to 3.0 | 0.533 |
| Respond to (versus ignoring) opportunities to  show compassion | 0.6 | -0.8 to 2.0 | 0.428 |

CI, confidence interval. Interaction term β Coefficients denote the difference in the β Coefficients between Blacks and non-Hispanic Whites. Interaction term β Coefficients > 0 indicate the association between the physician action and 5-item compassion measure score are stronger among Black individuals compared to non-Hispanic White individuals (reference). Interaction term β Coefficients < 0 indicate the association between the physician action and 5-item compassion measure score are stronger among non-Hispanic White individuals compared to Black individuals.

**Supplemental Figure 9**: Results of the pairwise univariable linear regression models testing for an interaction between physician actions not included in the final model (patient reported physician performed the action, “yes” versus “no/unsure”) and race (Black versus non-Hispanic White). The 5-item compassion measure is the dependent variable. β coefficients denote the difference in 5-item compassion measure score when the action is performed compared to when not performed. Horizontal lines are 95% confidence interval (solid = Black, dashed = non-Hispanic White).

| Actions | Interaction term β Coefficients | 95% CI | p-value |
| --- | --- | --- | --- |
|  |  |  |  |
| Introduce themselves | 6.2 | 2.1 to 10.2 | 0.003 |
| Face you when you were talking, instead of facing  the other way | 5.9 | 1.0 to 10.8 | 0.018 |
| Make and keep eye contact with you | 3.0 | -0.8 to 6.9 | 0.122 |
| Come close (within 3 feet) when talking to you,  instead of across the room | 2.6 | -1.1 to 6.2 | 0.165 |
| Ask your treatment preferences, and make  decisions with you, together | 1.7 | 0.3 to 3.0 | 0.018 |
| Ask about your worries (for example, "What  worries you the most?") | 1.3 | 0.1 to 2.4 | 0.038 |
| Ask how your daily functioning has been affected | 0.9 | -0.4 to 2.3 | 0.169 |
| Validate your emotions (for example, "This is hard  for you- anyone would feel upset") | 0.9 | -0.4 to 2.2 | 0.158 |
| Speak in a calm or soothing tone | 0.9 | -3.4 to 5.2 | 0.690 |
| Acknowledge that your personal experiences  influence your needs | 0.8 | -0.5 to 2.1 | 0.222 |
| Acknowledge and name your emotions (for  example, "You seem sad") | 0.7 | -0.3 to 1.7 | 0.187 |
| Give you supportive touch (for example, touch  your shoulder) | 0.7 | -0.4 to 1.7 | 0.208 |
| 19. Give you emotional support (for example, "I am  here with you") | 0.7 | -0.9 to 2.2 | 0.409 |
| 24. Understand how their words might affect you | 0.5 | -0.9 to 1.9 | 0.461 |
| 17. Grasp and explore clues that you need  compassion | 0.4 | -0.7 to 1.5 | 0.488 |
| 11. Express humor | 0.2 | -0.8 to 1.3 | 0.648 |
| 2. Sit down (versus stood up) while speaking with you | 0.2 | -0.9 to 1.2 | 0.724 |
| 10. Make "small talk" | -0.2 | -1.2 to 0.8 | 0.706 |

CI, confidence interval. Interaction term β Coefficients denote the difference in the β Coefficients between Blacks and non-Hispanic Whites. Interaction term β Coefficients > 0 indicate the association between the physician action and 5-item compassion measure score are stronger among Black individuals compared to non-Hispanic White individuals (reference). Interaction term β Coefficients < 0 indicate the association between the physician action and 5-item compassion measure score are stronger among non-Hispanic White individuals compared to Black individuals.
